# Editorial: Manganese-Enhanced MRI: A New Avenue of Functional and Structural Imaging in Neuroscience

**DOI:** 10.3389/fncir.2022.918500

**Published:** 2022-05-17

**Authors:** Makoto Osanai, Keigo Hikishima, Hirotaka Onoe

**Affiliations:** ^1^Laboratory for Physiological Functional Imaging, Division of Health Sciences, Department of Medical Physics and Engineering, Osaka University Graduate School of Medicine, Suita, Japan; ^2^Department of Physiology, Tohoku University Graduate School of Medicine, Sendai, Japan; ^3^Center for Information and Neural Networks (CiNet), National Institute of Information and Communications Technology, Osaka University, Suita, Japan; ^4^Medical Devices Research Group, Health and Medical Research Institute, National Institute of Advanced Industrial Science and Technology (AIST), Tsukuba, Japan; ^5^Human Brain Research Center (HBRC), Graduate School of Medicine, Kyoto University, Kyoto, Japan

**Keywords:** MRI, activity mapping, tract-tracing, brain structure, brain function, non-invasive imaging, calcium

To elucidate the functional circuitry underlying information processing in the brain, we need to monitor whole-brain activity changes when an animal displays a specific behavior or function or how these patterns are disturbed in neurological disorders. Therefore, reliable methods for analyzing neural activity throughout the brain are essential. Manganese-enhanced magnetic resonance imaging (MEMRI) and activation-induced manganese-enhanced MRI (AIM-MRI) have the potential to map whole-brain activity and identify structures related to a specific behavior, task, stimulus, operation, drug, or neuronal disease.

Mn^2+^ is a paramagnetic ion that enhances MRI contrast by shortening the longitudinal relaxation time (T1) of H^+^. In MEMRI, Mn^2+^ is used as a surrogate marker of Ca^2+^ influx, since it can enter neurons through Ca^2+^ channels. Mn^2+^ is taken up by mitochondria and other organelles and binds to proteins; thus, it is extruded very slowly from the cell (Almeida-Corrêa et al.; Deng et al.; Tanihira et al.; Figure 1A) and accumulates in neurons in an activity-dependent manner. Thus, MEMRI can visualize the history of neural activity non-invasively and enhance the contrast of brain structures.

**Figure 1:**
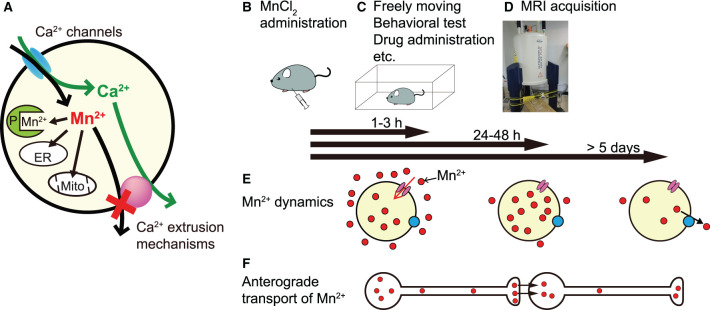
The principle and experimental sequence of MEMRI and Mn^2+^ dynamics in the brain. **(A)** Mn^2+^ enters the cell through Ca^2+^ channels and is difficult to extrude since it is incorporated in organelles, e.g., endoplasmic reticulum (ER) and mitochondria (Mito), and binds to various proteins (P). **(B–D)** An example experimental sequence of MEMRI (see text). **(E)** Mn^2+^ dynamics in the brain. Extracellular Mn^2+^ is increased rapidly within 1 h, remains high for 3 h (left), and returns to near control levels by 24 h (middle) after intraperitoneal MnCl_2_ injection. In the brain parenchyma, Mn^2+^ increases slowly, peaks at 24–48 h after administration (middle), and returns to control levels at 5 days (right) after a single administration. **(F)** Time course of the anterograde transport of Mn^2+^. The position of the transported Mn^2+^ (red particles) is roughly aligned with the time bar above **(E)**.

This Research Topic for Frontiers in Neural Circuits, which was planned to mark two decades after the first report of AIM-MRI (Lin and Koretsky, 1997), brings together the work of experts in functional and structural imaging using MEMRI. Here, we provide a summary of their contributions to this Research Topic.

## Original Research

### Mn^2+^ Dynamics in the Brain

In MEMRI, the usual experimental process is to administer MnCl_2_ (Figure 1B), allow the animals to move freely or impose a specific manipulation (Figure 1C), and perform an MRI scan (Figure 1D). To elucidate regions in which changes in neural activity are related to a specific task or manipulation and to standardize MEMRI protocols, the appropriate timing of that experimental sequence should be established. To resolve this issue, Tanihira et al. studied Mn^2+^ dynamics in the brain after systemic MnCl_2_ administration and clearly showed three time windows (Figure 1E). The first window, when Mn^2+^ concentration is increased in extracellular fluid, is at 1–3 h after its intraperitoneal administration; therefore, AIM-MRI records neural activity mainly during this period. The second window, when intracellular Mn^2+^ is maximal and extracellular Mn^2+^ is almost excreted, is at 24–48 h after administration, indicating the appropriate timing to record neural activity. The third window, when parenchymal Mn^2+^ concentration returns to near control values, is at least 5 days after single administration, indicating that one must wait at least >5 days to administer MnCl_2_ again for a repeat MEMRI experiment (note: Mn^2+^ dynamics in the brain depend on the species and the route of MnCl_2_ administration).

Mn^2+^ is also transported along axons and trans-synaptically to neighboring neurons in an activity-dependent anterograde manner, enabling direct monitoring of functional brain connectivity (Figure 1F). Almeida-Corrêa et al. showed how the activity state of neurons modulates interneuronal Mn^2+^ transport. They conducted two MRI scans at a 7-day interval and partially disrupted sensory input from the whiskers between the first and second scans. The differences in Mn^2+^ accumulation between the first scan after low-dose fractionated MnCl_2_ administration and the second scan without MnCl_2_ administration and with partial sensory deprivation provide evidence for neuronal activity-dependent accelerated Mn^2+^ transport to projection terminals and across synapses.

### Functional Mapping of Pain-Related Activity Using AIM-MRI

Arimura et al. and Inami et al. visualized pain-related activity using AIM-MRI. Arimura et al. reported the active brain regions associated with formalin-induced acute pain. They found pain-related neural activity in many of the projection regions of the sensory cortex as well as the sensory cortex. They also showed the combination of “designer receptors exclusively activated by designer drugs” (DREADD) and MEMRI can reveal the regions associated with pain-related brain activity and their causal relationships.

Inami et al. studied how whole-brain neural activity is changed by chronic neuropathic pain using a spared nerve injury model. They found chronic pain-related neural activity in not only the sensory cortex but also in regions related to cognition and emotion. Thus, they concluded that alterations of neural activity in areas that process pain cognition and emotion contribute to the chronification of neuropathic pain.

### MEMRI for Contrast Enhancement of Brain Structures

MEMRI can enhance the cytoarchitecture of brain tissues. Saito et al. applied MEMRI to evaluate hippocampal volume and to detect the abnormalities of hippocampal architecture induced by prenatal X-ray irradiation. They found abnormal hippocampal cytoarchitecture and volume reduction.

*Ex-vivo* MRI is a powerful tool to visualize detailed morphology of the brain since it avoids motion artifacts and scan time limitations. Sato et al. combined MEMRI and *ex-vivo* MRI to generate high-spatial-resolution microstructural brain images. They also evaluated the methods for *ex-vivo* MEMRI and showed a suitable protocol for imaging brain cytoarchitecture.

## Reviews of MEMRI

Saar and Koretsky and Deng et al. reviewed the use of MEMRI in neurodegenerative disease, and in ophthalmology and visual neuroscience, respectively. Sarr and Koretsky summarized the broad range of information obtained from MEMRI in many animal models of neurodegeneration and diseases with neurodegenerative components, and discussed the application of MEMRI in humans.

Deng et al. illustrated the various methods for visual neuroscience and presented the differences in the results. They also summarized the findings of MEMRI studies for neuroarchitecture evaluation, neuronal tract tracing, neural activity evaluation, and investigation of glial activity, and the limitations and future directions of MEMRI. This review is not only very useful for applying MEMRI to visual neuroscience but also contains a wealth of information that applies to other areas of neuroscience.

## Concluding Remarks

MEMRI is proving useful as a method to visualize the functional neuronal circuits and anatomy of the brain *in vivo*. Moreover, AIM-MRI makes it possible to record the history of neural activity throughout the brain of awake, freely moving animals, whereas blood-oxygen-level-dependent functional MRI can only record activity in the head-fixed condition. MEMRI can be used for non-invasive investigations of whole brain activity and neuronal connections, which do not depend on blood hemodynamics. MEMRI can be used to scan the same animals, enabling the execution of within-subject longitudinal studies, thus reducing the number of required subjects. To summarize, MEMRI has great potential for the study and diagnosis of various brain functions and neurological disorders, if we keep in mind some of the caveats exemplified in the articles of this Research Topic. We hope that this Research Topic will raise awareness of MEMRI not only in MRI specialists but also in neuroscience researchers.

## Author Contributions

MO, KH, and HO contributed to the inception, solicitation, drafting, and editing of this Research Topic and editorial.

## Funding

This work was supported by MEXT KAKENHI (JP17H05543, JP16H06276, JP21H03025, and JP21H03135 to MO) and Brain/MINDS [Mapping by Integrated Neurotechnologies for Disease Studies (JP19dm0207051, JP20dm0207051, and JP21dm0207115)], AMED (to MO).

## Conflict of Interest

The authors declare that the research was conducted in the absence of any commercial or financial relationships that could be construed as a potential conflict of interest.

## Publisher's Note

All claims expressed in this article are solely those of the author and do not necessarily represent those of their affiliated organizations, or those of the publisher, the editors and the reviewers. Any product that may be evaluated in this article, or claim that may be made by its manufacturer, is not guaranteed or endorsed by the publisher.
